# Effects of different phosphorus and potassium supply on the root architecture, phosphorus and potassium uptake, and utilization efficiency of hydroponic rice

**DOI:** 10.1038/s41598-024-72287-1

**Published:** 2024-09-11

**Authors:** Ya Liu, Jiping Gao, Yanze Zhao, Yichen Fu, Bingchun Yan, Xue Wan, Guoqing Cheng, Wenzhong Zhang

**Affiliations:** Agronomy College, Shenyang Agriculture University, Shenyang, 110086 China

**Keywords:** Rice, Root morphology, Phosphatase, Dry matter, P and K uptake and utilization, Yield and its components, Physiology, Plant sciences

## Abstract

Phosphorus (P) and potassium (K) affect seedling growth, root configuration, and nutrient uptake in hydroponic rice, but there are few studies on all growth stages of rice. The purpose of this experiment was to determine the response characteristics of root morphology, plant physiology, and P and K uptake and utilization efficiency to different supplies of P and K. Two local conventional rice varieties (Shennong 265 and Liaojing 294) were used as experimental materials across four treatments, including HPHK (sufficient P and K supply), HPLK (sufficient P supply under low K levels), LPHK (sufficient K supply under low P levels) and LPLK (low P and K levels) in a hydroponic setting. The results showed that HPHK and HPLK significantly decreased the acid phosphatase activity of leaves and roots from full heading to filling stages when compared to LPHK and LPLK. Sufficient supply of P or K significantly increased the accumulation of P and K (aboveground, leaves, stem sheath, and whole plant) and root morphological parameters (root length, root surface area, total root volume, and tips) during major growth stages when compared to LP or LK levels. HPHK was significantly higher than other treatments in terms of dry weight and the root activity at the main growth stage, P and K uptake rates in nutrient solutions at various stages, related P and K efficiency at the maturity stage, yield, effective panicle number, and grain number per panicle. In addition, the effect of HPHK on the above indexes were significantly greater than those of single sufficient supply of P or K. In conclusion, HPHK can improve plant configuration, increase plant P and K absorption and root activity, and increase rice yield and related P and K utilization efficiency.

## Introduction

Rice is one of the main food crops in the world, and balanced fertilization with nitrogen, phosphorus and potassium (NPK) can effectively improve grain and milled rice yields^1,2^. Nutrient deficiency (one or more) can affect plant growth, physiology, and biochemistry characteristics^3^. Therefore, the maintenance of nutrient balance in plants is of great significance to promote crop yield and improve efficient nutrient use^4^. However, the interaction between NPK, especially the specific mechanism of P and K to promote crop yield, is not yet clear. In addition to nitrogen N, P and K, essential nutrients required by crops, participate in many physiological and biochemical processes in plants, including material accumulation, production of photosynthetic products, activation of key enzyme activities, regulation of stomatal movement, and cell osmotic pressure, while also influencing photosynthetic mechanisms, including PSI and PSII^5–8^.

The concentration of P and K in the environment affects root morphology and related physiological metabolism^9,10^. The different characteristics of root morphology and physiological are closely related to nutrient uptake and utilization^11^. The dependence of maize on ACP activity and root parameters can represent the ability of plant to tolerate low P to a certain extent: the lower the dependence, the worse the ability to tolerate low P^12^. Under suitable P supply conditions, wheat dry weight was positively correlated with P accumulation, total root length, total root volume and total root surface area, while low P would increase wheat root parameters (such as total root length and number of root tips)^13^. Under P deficiency condition, root growth, specific root length and root length density decrease significantly, and root ACP activity change correspondingly^14–16^. P deficiency significantly affects root metabolic activity, increases root ACP activity, and decreases grain yield^17,18^. While K is less important than P for plant root configuration, it still influences root configuration^10^. K-deficiency significantly reduces root dry weight, total root length, and root length density^19,20^. The reasonable regulation of K transport and distribution in plants and the maintenance of high root K absorption capacity, root activity (RA), root–shoot ratios, and root length density are conducive to improving the efficiency of crop K absorption^21,22^.

P and K concentrations in the environment affect plant growth, yield, nutrient uptake, and utilization^23,24^. Plant configuration (including plant height, leaf area, and root morphology) under different P and K supply conditions affect P and K uptake in various organs, further affecting yield and the associated P and K utilization. In terms of plant growth and yield, plant P deficiency significantly decreases cotton P metabolism and root configuration, affects rice plant configuration (including dry weight, plant height, and tillering number), and inhibits dry matter accumulation in both crops^25,26^. Plant K deficiency inhibits root dry weight and configuration at the seedling stage of rice, and the plant height and yield components of wheat^27,28^. In addition, in terms of the associated P and K efficiency, a P-deficient supply increases P absorption per unit root length (PuRL) and P absorption per unit root weight (PuRW)^14^. Suitable P levels can promote the increase of rice yield, improve the yield’s composition, and increase the plant N utilization rate^29^. The accumulation and transport of P in rice affect the efficiency of external P acquisition and internal P efficiency^30^. On the other hand, suitable K levels increase K accumulation, root dry weight, and N and P absorption of rice plants, but decrease the internal K efficiency (KIE), the K agronomic efficiency (KAE), and the K physiological efficiency (KPE)^31,32^. K deficiency decreases the K absorption, accumulation, and utilization efficiency of wheat, and increases the K transport rate (KTE) of cotton^33^. In addition, the nutrient absorption efficiency in solution can directly reflect the nutrient absorption intensity of plants in a hydroponics test.

However, in hydroponic experiments, the effects of low P (K) supply on plant nutrient uptake and root morphology were mostly studied at the seedling stage. There are few studies on the effects of the different supply of P and K (sufficient or lacking) on root morphology and related physiological characteristics, the yield, and the P and K efficiency of hydroponic rice during all growth stages. Therefore, using Shennong265 and Liaojing294 as experimental materials and root morphology as the entry point, P and K efficiency and plant physiological changes in the ‘aboveground—root-nutrient solution’ system of hydroponic rice during growth stages at different P and K supplies were compared and analysed. The aim of this study was to further clarify the role of P and K interactions in the nutrient uptake in rice roots, and the causes of yield and the associated physiological mechanism differences.

## Materials and methods

### Plant growth conditions and experimental treatments

Hydroponic experiments were conducted in May 2022 at the Kalima Rice Experiment Station (41°12′E, 122°23′E) in Shenyang Agricultural University, Liaoning Province, China. The two rice varieties, Shennong265 (SN265) and Liaojing294 (LJ294), were local japonica conventional varieties with similar growth durations. Four treatments were set up in this experiment, which included HPHK treatment (sufficient supply of P and K: 10 mg/L of P and 40 mg/L of K), HPLK treatment (sufficient supply of P under low K levels: 10 mg/L of P and 2 mg/L of K), LPHK treatment (sufficient supply of K under low P levels: 0.5 mg/L of P and 40 mg/L of K) and LPLK treatment (low P and K levels: 0.5 mg/L of P and 2 mg/L of K). Except for P and K, the concentrations of other elements were configured according to the formula of the International Rice Research Institute (IRRI). These concentrations were: 40 mg/L of N, Ca, and Mg, 5.6 mg/L of Si, 2 mg/L of Fe, 0.5 mg/L of Mn, 0.2 mg/L of B, 0.05 mg/L of Mo, 0.01 mg/L of Zn and Cu. The two rice varieties were cultivated from seed to a 4-leaf age (30 days after sowing) and then transplanted into hydroponic pots, with two plants per hole and two holes per pot. 7 L of nutrient solution was added to each pot to ensure that the root of each plant was completely immersed in the nutrient solution. The nutrient solution was changed every 10 days and hydroponic pots were randomly relocated to avoid positional differences. The pH of the nutrient solution was adjusted to 5.0 daily using 1 mol/L of HCl or 1 mol/L of NaOH, and deionized water was replenished promptly to ensure the total volume of the solution in the hydroponic pots. Two weeks before harvest, the nutrient solution in the hydroponic pots was replaced with deionized water of the same volume and pH. In this study, six pots were sampled per treatment, per variety, per period, with a total of 192 pots. Each indicator was measured three times for statistical analysis.

### Yield and yield components

At the maturity stage, the theoretical yield was calculated according to the yield component factors. Yield components included effective panicle number, seed setting rate, 1000-grain weight, and number of grains per panicle. The grains of each plant were divided into filled and unfilled grains, and the 1000-grain weight (the total weight of 1000 filled grains), and the seed setting rate (the ratio of the number of filled grains to the total number of grains) were calculated. In addition, the ear-bearing tillering rate was also measured.

### Determination of acid phosphatase (ACP) activity and RA

In order to study the physiological properties of leaves and roots, six-hole plants at each growth stage (tillering, full heading, filling, and maturity stages) were sampled Roots were cleaned with deionized water and excess water was removed with filter paper, leaves and roots were then collected, snap-frozen with liquid N for 5 min, and then finally transferred to an ultra-low-temperature refrigerator at − 80 °C for storage. ACP was determined using the P-nitrophenyl phosphate (pNPP) microplate method. Leaf and root tissues (0.1 g) were weighed and ground into a slurry in an ice bath by adding 1 mL of buffer solution (pH 4.8). The slurry was centrifuged at 8000*g* for 10 min at 4 °C, and the supernatant was extracted into a new centrifuge tube which was placed on ice. The subsequent measurements were conducted in accordance with manufacturer instructions for each kit (Shanghai yuanye Bio-Technology Co., Ltd.). The root tissue (0.1 g) was weighed and the RA was determined according to the manufacturer instructions for the plant root activity detection kit (Beijing solarbio science and technology Co., Ltd.).

### Determination of plant dry matter, and P and K concentration

Three-hole plants per treatment were collected at the tillering, full heading, filling, and maturity stages to determine plant height. The cleaned plants were divided into four parts: stem sheath, leaf, panicle, and root, desiccated at 105 °C for 0.5 h, and then dried at 80 °C until achieving a constant weight. Notably, the above-ground part of the tillering stage was divided into stem sheath and leaf. The dry weight of each part and the root–shoot ratios were calculated. At the same stage, the dry samples of each part were crushed. Dry samples (0.5 g) were weighed and processed using the sulfuric acid-hydrogen peroxide wet digestion method. The extracted solution was diluted to 100 mL for the determination of P and K concentration. The vanadium molybdenum yellow method was used to determine the P concentration, and the flame photometer was used to determine the K concentration of plants^34,35^. LPC (LKC), SPC (SKC), PPC (PKC), RPC (RKC) and APC (AKC) represented leaf, stem sheath, panicle, root, and aboveground P (K) concentrations, respectively. LPA (LKA), SPA (SKA), PPA (PKA), RPA (RKA), APA (AKA), and TPA (TKA) represented P (K) accumulation in leaves, stem sheath, panicle, root, aboveground part, and whole plant, respectively. Among these, APA (AKA) was the sum of P (K) accumulation in the leaves, stem sheath, and panicle, and TPA (TKA) was the sum of P (K) accumulation in the aboveground and root. APC (AKC) was the ratio of APA (AKA) to its corresponding dry weight.

### Determination of root morphological parameters

During the same stages, three-hole plants per treatment were collected and placed on a plastic plate containing deionized water to measure root morphological parameters. The perfect V700 root scanner was used to capture root morphology pictures. The Win-RHIZO PRO 2013 root analysis software was used to comprehensively analyse the root length, root surface area, AD, tap volume, and tips for each treatment. Moreover, specific root length, STD, root tissue density, and specific surface area were calculated. The relevant plant dry weight and root indicator calculation formulas and abbreviations are shown in Table 1.

**Table 1:** Indicators related to plant dry weight, root parameters, leaf and root phosphatase activity.

Abbreviations	Definition	Units
DMAbove or DMRoot	Aboveground or root dry weight	g/hole
DMPlant	Sum of aboveground and root dry weight	g/hole
Root length	Total root length	cm
SRL	Specific root length. Ratio of total root length to root dry weight	cm/g
SRSA	Specific root surface area. Ratio of root surface area to root dry weight	cm^2^/g
Tap volume	Main root volume	cm^3^
RTD	Root tissue density. Ratio of root dry weight to main root volume	g/cm^3^
Tips	Total number of plant root tips	number/10^3^
STD	Ratio of root tip number to root dry weight	tips/g/10^3^
PuRL (KuRL)	Ratio of plant P (K) accumulation to total root length	mg/m
PuRW (KuRW)	Ratio of plant P (K) accumulation to root dry weight	mg/g
RA	Root activity	μg ·α-NA·FW/(g· h)
AD	Average root diameter	mm
[ACP]leave or [ACP]root	Acid phosphatase activity of leave or root	nmol/g/min
Root–shoot ratio	Ratio of root to aboveground dry weight	

### Measurement of the P and K uptake rate in nutrient solutions

Before replacing the nutrient solution, 10 mL of water samples were collected every 10 days, and 3 replicates were collected for each treatment. 5 mL of filtered water samples were selected and the residual P concentration in nutrient solutions were determined by ammonium molybdate spectrophotometry, while the residual K concentration were determined by flame spectrophotometry^36,37^. The P and K uptake rates (PUR and KUR) were measured in the nutrient solutions every 10 days. The calculation formula was:

$${\text{PUR}}\;({\text{KUR}})\;({\text{mg/d}}) = \left( {{\text{OPC}}\;\left( {{\text{OKC}}} \right){\text{ }} - {\text{ RPC}}\;\left( {{\text{RKC}}} \right)} \right) \times 7\;{\text{L/}}10\;{\text{days}}.$$

OPC (OKC) and RPC (RKC) represent the initial P (K) concentration in the nutrient solution and the residual P (K) concentration in the nutrient solution, respectively.

### Data analysis

Analysis of variance (ANOVA) was performed using the SPSS26.0 statistical software and Origin2021PRO was used for graphing. The relevant P (K) efficiency calculation formulas and abbreviations are shown in Table 2.

**Table 2:** Plant P (K) related utilization efficiency.

Abbreviations	Definition	Units
PAI (KAI)	P (K) absorb intensity. The ratio of the difference in plant P (K) accumulation between adjacent fertility periods to time	mg/(m^2^·d)
PIE (KIE)	P (K) internal uptake efficiency. Ratio of above-ground dry weight to plant P (K) accumulation	kg/kg
PAE (KAE)	P (K) agronomic efficiency. Ratio of seed yield to P (K) application	kg/kg
PUE (KUE)	P (K) uptake efficiency. The ratio of plant P (K) accumulation to P (K) application	%
PPE (KPE)	P (K) physiological efficiency. Ratio of grain yield to plant P (K) accumulation	kg/kg
GPR (GKR)	P (K) requirements for 100 kg grain. 100 times ratio of plant P (K) accumulation to grain yield	kg· P (K)·100 kg
PHI (KHI)	P (K) harvest index. 100 times ratio of seed P (K) to plant P (K) accumulation	%
ETR	Earbearing tiller rate. Ratio of effective number of tillers to maximum number of tillers at maturity	%
PUEb (KUEb)	P (K) dry matter production efficiency. The ratio of plant dry weight to P (K) accumulation	kg/kg· P (K)
PTE (KTE)	P (K) translocation rate. Ratio of the difference between the P (K) accumulation of nutrient organs at the full heading and maturity stage, and the P (K) accumulation of nutrient organs at the full heading stage	%

## Results

### Rice plant architecture and yield components

**Fig. 1:**
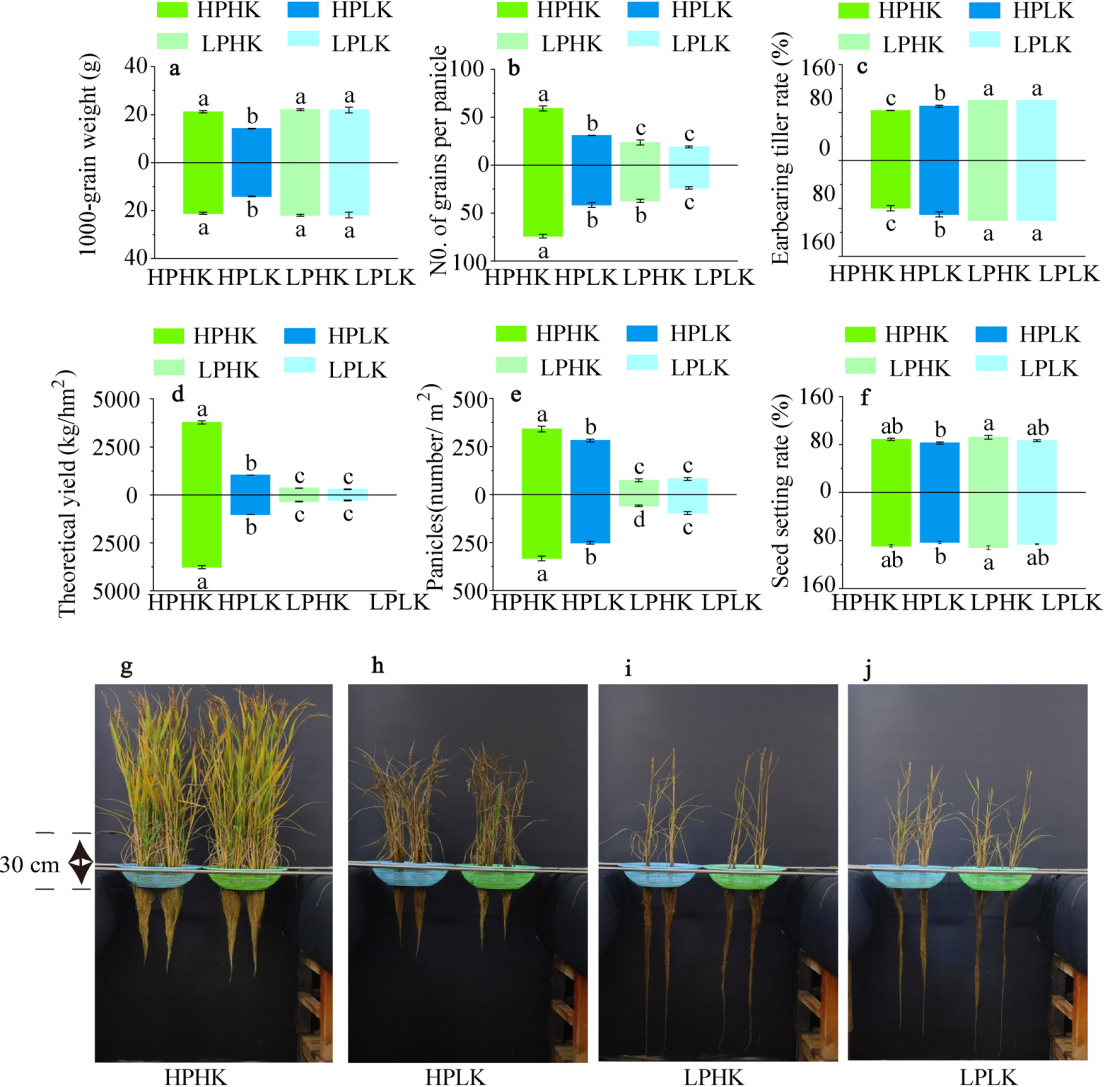
Rice yield and plant architecture in different P and K nutrient solutions. Theoretical yield and its components (**a**–**f**). Plant architecture at harvest stage, in which the blue and green hydroponic pots represent SN265 and LJ294, respectively (**g**–**j**). The data above the origin of the Y-axis represents SN265, and the data below represents LJ 294. The four treatments were HPHK (10 mg/L P and 40 mg/L K), HPLK (10 mg/L P and 2 mg/L K), LPHK (0.5 mg/L P and 40 mg/L K) and LPLK treatment (0.5 mg/L P and 2 mg/L K), respectively. The error bar represents ± standard deviation. Different lowercase letters indicate statistical significance at P < 0.05 level.

The patterns of change in plant architecture and yield components were similar between the two rice varieties under different P and K nutrient solutions. The plant architectures of the two varieties under HPHK treatment were better than those of other treatments, while those treated with HPLK were better than those treated with low P levels. There were negligible differences in plant architectures between LPHK and LPLK treatments (Fig. 1g–j).

Compared to other treatments, HPHK had larger theoretical yields, with increases of 2.69 to 12.90 times that of the two varieties (Fig. 1d). In terms of yield components, compared to other treatments, the number of effective panicles under HPHK were significantly increased between 0.21 and 4.63 times that of the two varieties (Fig. 1e); the number of grains per panicle was significantly increased between 0.78 and 2.12 times (Fig. 1b); while the ear-bearing tiller rate was significantly reduced (Fig. 1c). In addition, the seed setting rate and 1000-grain weight of the two varieties under HPLK treatment were lower than those of other treatments. Among HPHK, LPHK, and LPLK treatments, there are not significant differences between seed setting rates and the 1000-grain weights (Fig. 1a,f).

### ACP activities of leaves and roots

The change patterns in ACP activity of roots and leaves of the two varieties were similar under different P and K treatments. Compared to Low K levels (LK), the ACP activity of leaves and roots at Low P (LP) levels changed more substantially. Compared to LP, sufficient P supply significantly decreased the ACP activity of leaves from full heading to filling stages, as well as the ACP activity of roots at each growth stage.

**Fig. 2:**
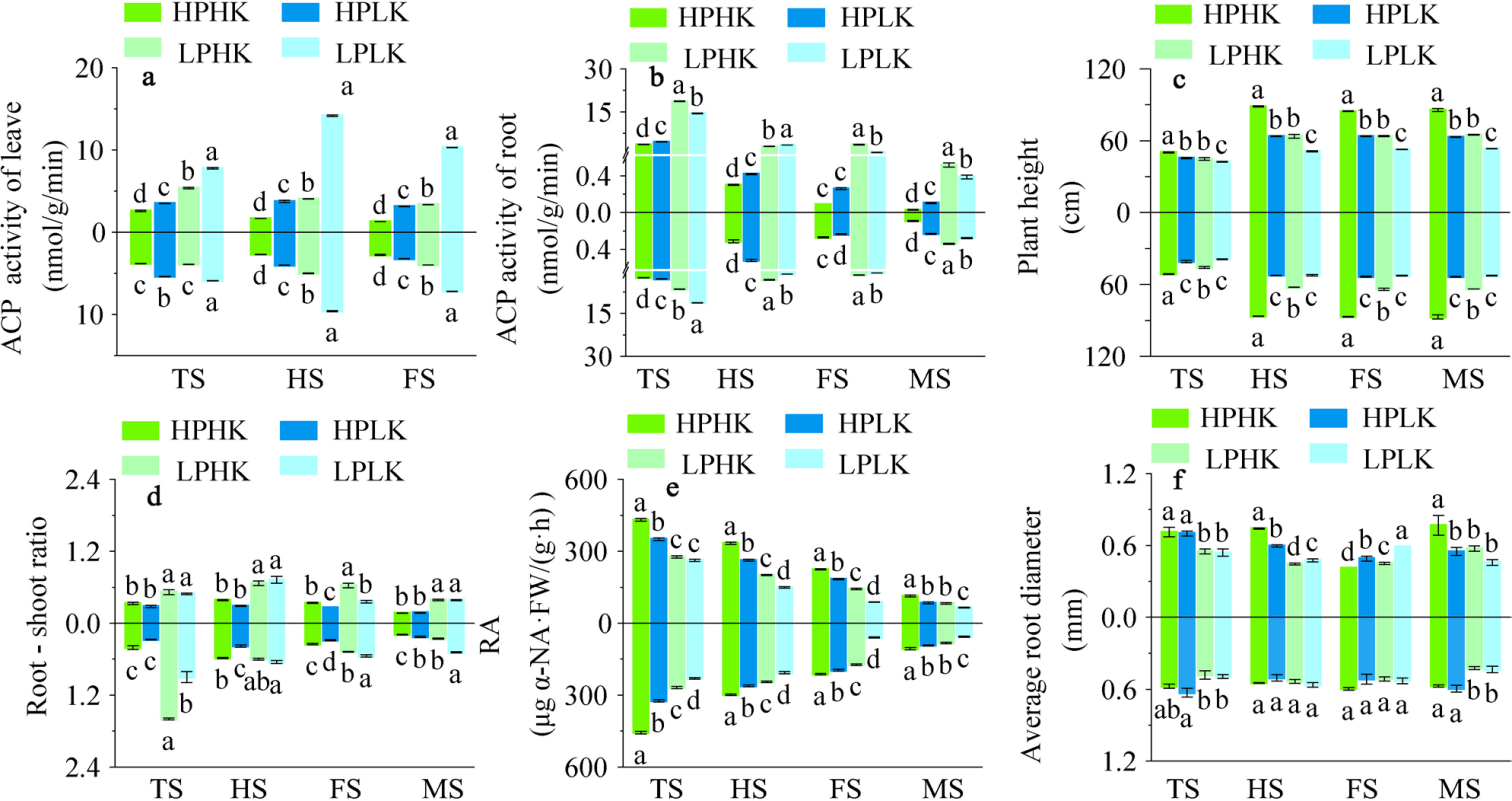
Leaf and root ACP activities, plant height, root to shoot ratio, RA, average root diameter in different P and K nutrient solutions. Leaf ACP (**a**), root ACP (**b**), plant height (**c**), root–shoot ratio (**d**), RA (**e**), and average root diameter (**f**). TS, HS, FS and MS represent tillering stage, full heading stage, filling stage and maturity stage respectively. The data above the origin of the Y-axis represents SN265, and the data below represents Liaojing 294. The four treatments were HPHK (10 mg/L P and 40 mg/L K), HPLK (10 mg/L P and 2 mg/L K), LPHK (0.5 mg/L P and 40 mg/L K) and LPLK treatment (0.5 mg/L P and 2 mg/L K), respectively. The error bar represents ± standard deviation. Different lowercase letters indicate statistical significance at P < 0.05 level.

The ACP activity of leaves from full heading to filling stages, and of roots at the tillering, full heading, and maturity stages under HPHK treatment were significantly lower than those under HPLK. Compared to HPLK, LPHK, and LPLK, the ACP activity of the two leaf varieties under HPHK treatment decreased between 14.48 and 88.29% (Fig. 2a), while the root ACP activity decreased between 14.53 and 94.34% (Fig. 2b). In addition, compared to LP, the ACP activity of the two leaf varieties under HPLK treatment decreased between 7.10 and 73.48%, while the root ACP activity decreased between 15.99 and 87.88% (Fig. 2a,b).

### Plant height, root–shoot ratio, root diameter, and RA

In terms of plant height, RA, and AD, the two rice varieties showed similar patterns of change under different P and K nutrient solutions. With the progression of the growth period, the root–shoot ratio increased first and then decreased, while the RA decreased gradually, with both indexes finally reaching their lowest at the maturity stage.

Compared to HPLK, LPHK, and LPLK, HPHK significantly increased plant height and RA at each growth stage. Among these, the plant height increase of the two varieties under HPHK was between 31.91 and 73.43% from full the heading to the maturity stages (Fig. 2c). At each growth stage, the RA of the two rice varieties under HPHK treatment significantly increased between 8.10 and 257.30%, respectively (Fig. 2e). In addition, the plant height of the two varieties under LPHK treatment increased between 0.16 and 24.93% when compared to LK (Fig. 2c). At the same P level, sufficient K supply also increased the RA, albeit to a lesser extent. This indicated that a sufficient P and K supply could significantly increase rice RA, but that the impact of P on RA was much larger than that of K (Fig. 2e).

Compared to LP, HPHK treatment significantly reduced the root–shoot ratio of SN265 at the tillering, full heading, and maturity stages between 33.12 and 56.41%, and those of LJ294 at the tillering, filling, and maturity stages between 26.27 and 74.57%. There was no significant change in the root–shoot ratios between HPHK and HPLK treatments (Fig. 2d). In addition, the average root diameter of SN265 and LJ294 under HPHK treatment increased by 31.63% and 67.75% (P < 0.05) at the maturity stage when compared to LP, respectively (Fig. 2f).

### Dry matter accumulation in different organs

**Fig. 3:**
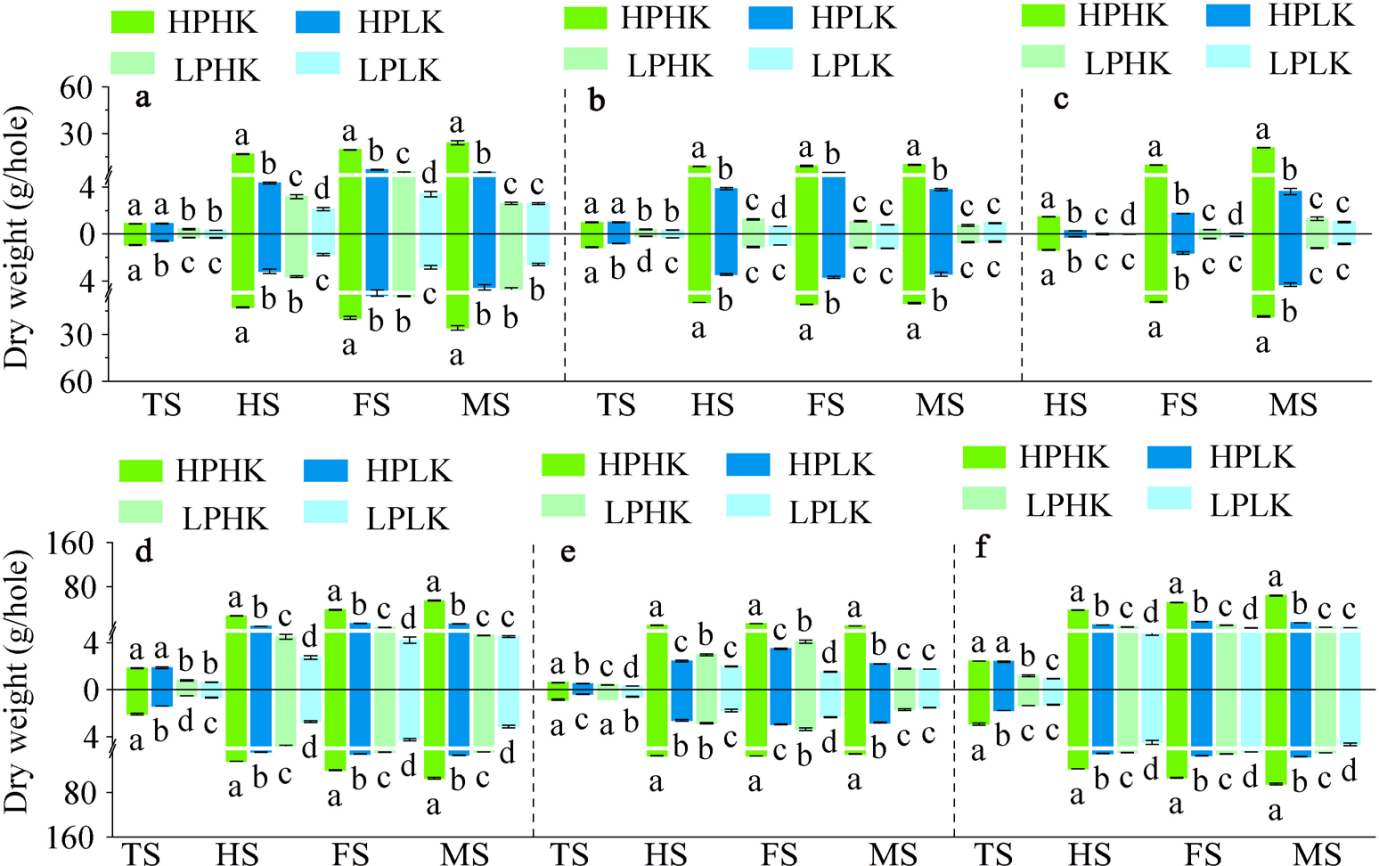
Dry matter accumulation of plants in different P and K nutrient solutions. Stem sheath dry weight (**a**), leaf dry weight (**b**), panicle dry weight (**c**), aboveground dry weight (**d**), root dry weight (**e**) and dry weight per hole (**f**). TS, HS, FS and MS represent tillering stage, full heading stage, filling stage and maturity stage respectively. The data above the origin of the Y-axis represents SN265, and the data below represents Liaojing 294. The four treatments were HPHK (10 mg/L P and 40 mg/L K), HPLK (10 mg/L P and 2 mg/L K), LPHK (0.5 mg/L P and 40 mg/L K) and LPLK treatment (0.5 mg/L P and 2 mg/L K), respectively. The error bar represents ± standard deviation. Different lowercase letters indicate statistical significance at P < 0.05 level.

With the progression of the growth period, the dry weight of the stem sheaths, leaves, and panicles of the two rice varieties showed a gradual increasing trend (Fig. 3a–c). Compared to HPLK, LPHK, and LPLK, HPHK treatment significantly increased the above indicators of the two varieties from the full heading to the maturity stages. Among these, HPHK treatment increased stem sheath dry weight between 1.88 and 8.94 times (Fig. 3a), leaves between 0.83 and 14.44 times (Fig. 3b), and panicles between 3.30 and 161.47 times (Fig. 3c).

With the progression of the growth period, the DMabove of the two rice varieties showed a gradual increasing trend, while the DMroot showed an initial increasing and then decreasing trend. From the full heading to the maturity stages, the DMabove and DMplant of both varieties reached a maximum under HPHK treatment, followed by HPLK. Moreover, this was significantly higher for HPHK treatment when compared to other treatments, while HPLK treatment was significantly higher than LP treatment (Fig. 3d,f). Compared to other treatments, the increase in DMabove of the two rice varieties under HPHK treatment ranged between 1.84 and 16.29 times, and the increase in DMplant ranged between 2.03 and 12.84 times. Compared to other treatments, HPHK significantly increased the DMroot of the two rice varieties from the full heading to the maturity stages, ranging between 2.24 and 7.74 times (Fig. 3e).

### P and K concentrations in different organs

**Fig. 4:**
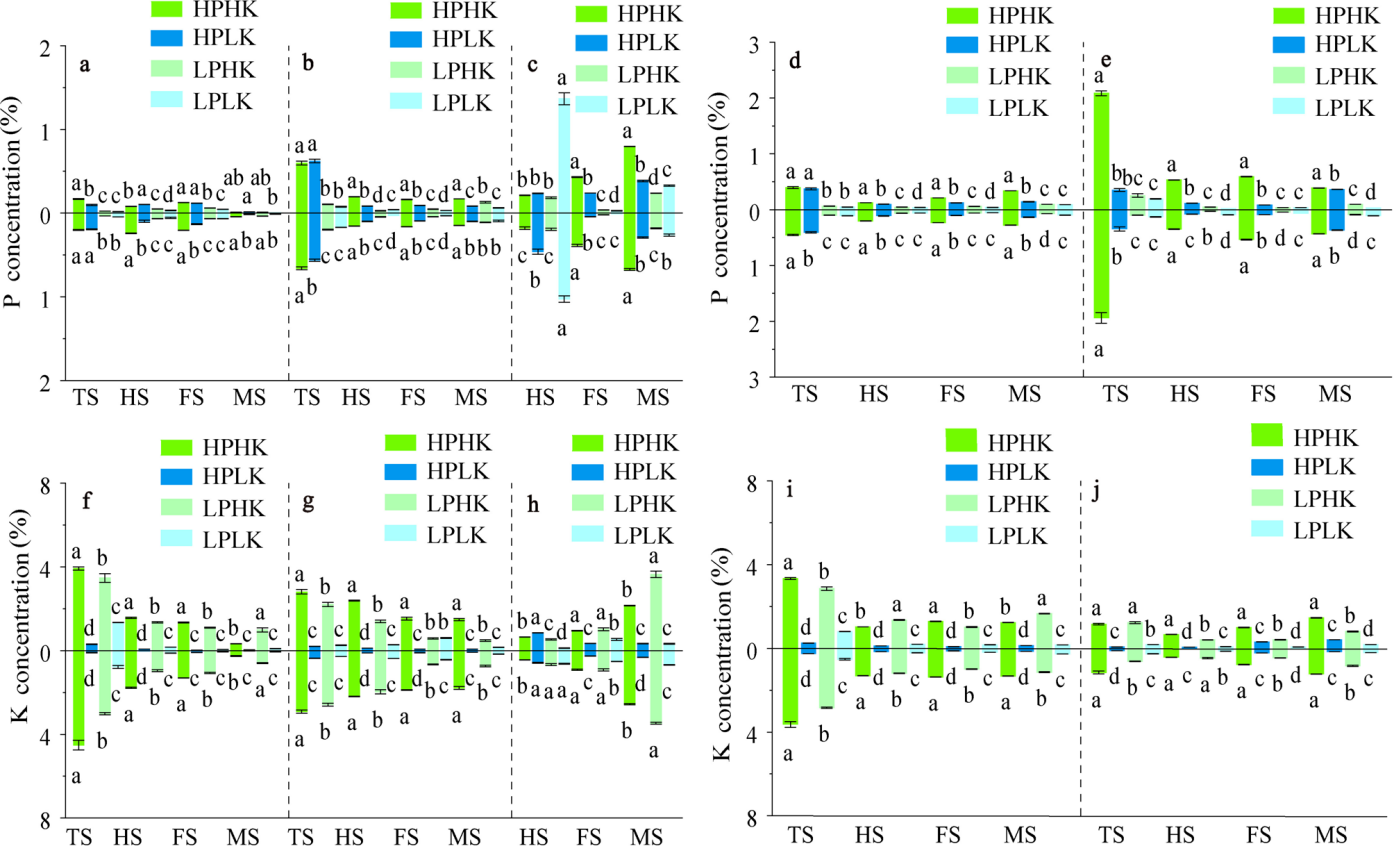
P and K concentrations of different organs in different P and K nutrient solutions. Stem sheath P (**a**), leaf P (**b**), panicle P concentration (**c**), aboveground P (**d**), root P concentration (**e**), stem sheath K (**f**), leaf K (**g**), panicle K concentration (**h**), aboveground K (**i**) and root K concentration (**j**). TS, HS, FS and MS represent tillering stage, full heading stage, filling stage and maturity stage respectively. The data above the origin of the Y-axis represents SN265, and the data below represents Liaojing 294. The four treatments were HPHK (10 mg/L P and 40 mg/L K), HPLK (10 mg/L P and 2 mg/L K), LPHK (0.5 mg/L P and 40 mg/L K) and LPLK treatment (0.5 mg/L P and 2 mg/L K), respectively. The error bar represents ± standard deviation. Different lowercase letters indicate statistical significance at P < 0.05 level.

As shown in Fig. 4, from the tillering to the filling stages, the SPC and LPC of the two varieties with sufficient P supply were significantly higher than those of LP. At the maturity stage, the order of the LPC of the two rice varieties among treatments was: HPHK > LPHK > HPLK > LPLK. Compared to LP, from the tillering to the filling stages, the SPC of the two varieties under HPHK treatment were significantly increased between 0.57 and 7.72 times, respectively (Fig. 4a); while the LPC were significantly increased between 2.37 and 6.96 times, respectively (Fig. 4b). The PPC of the two rice varieties under sufficient P supply was significantly higher than those of LP at the filling and maturity stages (Fig. 4c). In addition, HPHK treatment significantly increased the LPC from the full heading to the maturity stages, and the PPC from the filling to the maturity stages of the two varieties when compared to HPLK (Fig. 4b,c). However, the SPC showed no significant pattern between HPHK and HPLK treatments (Fig. 4a).

Compared to LP, sufficient P supply effectively increased the APC at all stages, and the RPC at the tillering, filling, and maturity stages of the two rice varieties. Among these, the APC under HPHK treatment was significantly increased between 1.74 and 7.29 times, respectively (Fig. 4d), and the RPC was significantly increased between 2.94 and 18.62 times respectively (Fig. 4e). In addition, compared to HPLK, HPHK treatment significantly increased the APC and RPC of the two rice varieties from the full heading to the maturity stages (Fig. 4d,e).

As shown in Fig. 4, sufficient K supply significantly increased the SKC of the two varieties during all growth stages, the LKC at the tillering, full heading, and maturity stages, and the PKC from the filling to the maturity stages when compared to LK. Among these, the SKC of the two rice varieties under HPHK treatment significantly increased between 1.92 and 86.44 times, respectively (Fig. 4f); the LKC significantly increased between 4.79 and 21.66 times, respectively (Fig. 4g); and the PKC significantly increased between 0.74 and 6.90 times, respectively (Fig. 4h). In addition, HPHK treatment significantly increased the SKC from the tillering to the filling stages, and the LKC during all growth stages, but significantly decreased the PKC at the maturity stage when compared to LPHK (Fig. 4f–h).

Sufficient K supply significantly increased the AKC and RKC of the two rice varieties during all growth stages when compared to LK (Fig. 4i,j). Among these, the AKC of the two varieties under HPHK treatment was significantly increased between 3.18 and 13.84 times (Fig. 4i), respectively; and the RKC was significantly increased between 2.19 and 18.06 times (Fig. 4j), respectively. Moreover, HPHK treatment significantly increased the AKC at the tillering and filling stages, and significantly increased the RKC at the filling and maturity stages when compared with LPHK.

### P and K accumulation in different organs

**Fig. 5:**
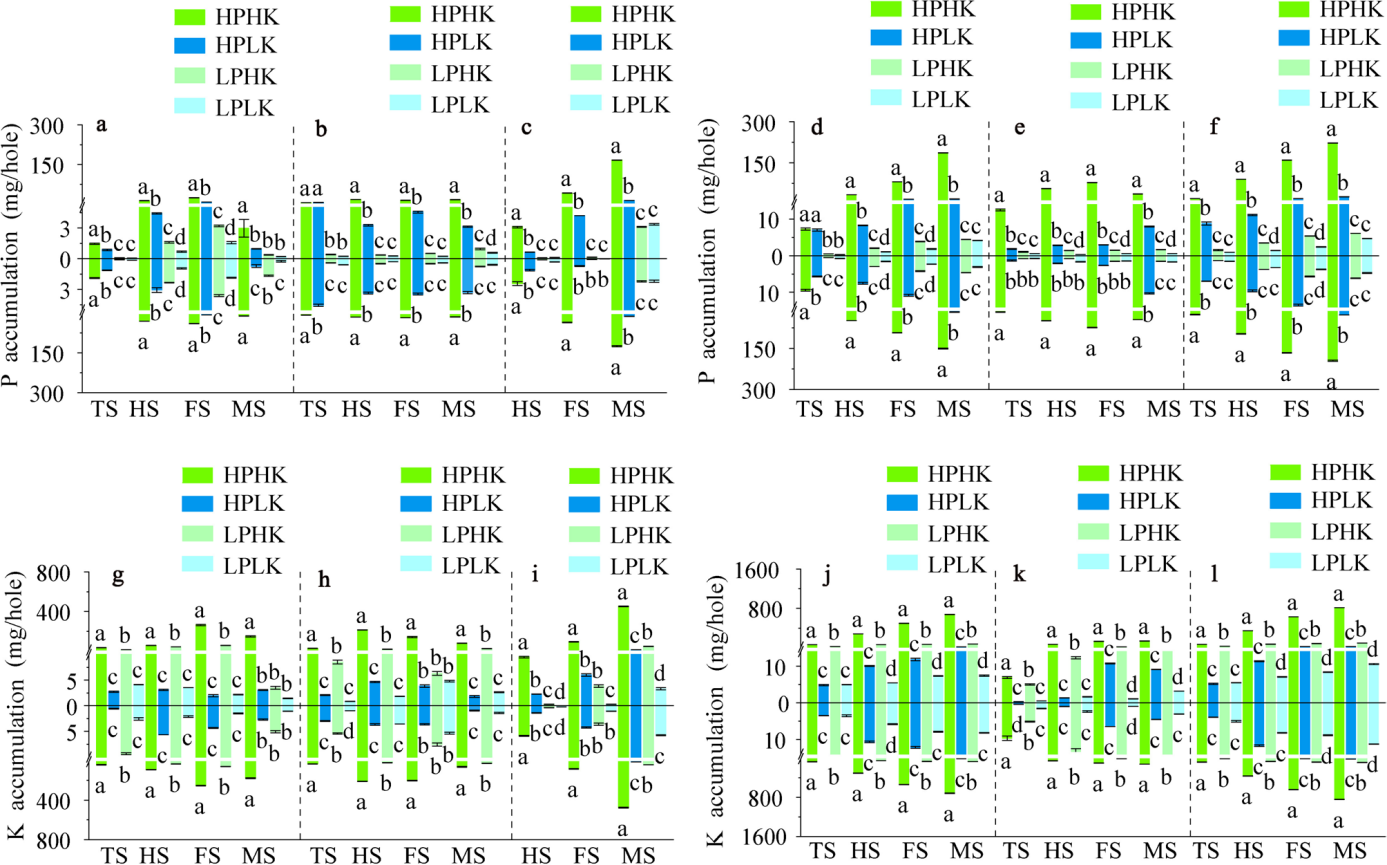
P and K accumulation of different organs and PUR (KUR) under different P and K nutrient solutions. P and K accumulation of different organs (**a**–**l**). Stem sheath P accumulation (**a**), leaf P accumulation (**b**), panicle P accumulation (**c**), aboveground P accumulation (**d**), root P accumulation (**e**), P accumulation per hole (**f**), stem sheath K accumulation (**g**), leaf K accumulation (**h**), panicle K accumulation (**i**), aboveground K accumulation (**j**), root K accumulation (**k**) and K accumulation per hole (**l**). TS, HS, FS and MS represent tillering stage, full heading stage, filling stage and maturity stage respectively. The data above the origin of the Y-axis represents SN265, and the data below represents Liaojing 294. The four treatments were HPHK (10 mg/L P and 40 mg/L K), HPLK (10 mg/L P and 2 mg/L K), LPHK (0.5 mg/L P and 40 mg/L K) and LPLK treatment (0.5 mg/L P and 2 mg/L K), respectively. The error bar represents ± standard deviation. Different lowercase letters indicate statistical significance at P < 0.05 level.

As shown in Fig. 5, from the tillering to the maturity stages, as the growth progressed, SPA and RPA under each treatment showed an increasing and then decreasing trend, while APA and TPA showed a gradual increasing trend and reached a maximum at the maturity stage (Fig. 5a,d–f).

Compared to LP, sufficient P supply significantly increased SPA of both varieties from the tillering to the filling stages, and LPA during all whole growth stages. Meanwhile, sufficient P supply also significantly increased PPA of SN265 during all growth stages, and LJ294 at the full heading and maturity stages. In addition, HPHK treatment was significantly higher than HPLK from the full heading to the maturity stages. Among these, compared to LP, SPA and LPA were significantly increased between 6.68 and 30.81 times and between 11.77 and 65.04 times, respectively (Fig. 5a,b), while PPA at the maturity stage was significantly increased between 48.66 and 55.67 times (Fig. 5c).

Sufficient P supply significantly increased APA and TPA in both rice varieties when compared to LP, while HPHK treatment was significantly higher than HPLK from full the heading to the maturity stages (Fig. 5d,f). RPA of both varieties reached a maximum under HPHK treatment, followed by HPLK, during all growth stages (Fig. 5e). Moreover, this was significantly higher under HPHK treatment when compared to other treatments, while the HPLK treatment of SN265 was significantly higher than LP. Compared to LP, APA, RPA, and TPA in both varieties under HPHK treatment significantly increased between 11.73 and 47.89 times, between 11.14 and 160.21 times, and between 11.85 and 65.41 times, respectively.

From the tillering to the maturity stages, SKA and LKA of the two rice varieties under each treatment showed an increasing and then decreasing trend as growth progressed (Fig. 5g,h). Compared to LK, sufficient K supply significantly increased SKA from the tillering to the filling stages, LKA at the tillering, full heading, and maturity stages, and PKA at the maturity stage in both varieties. Compared to LK, SKA, LKA, and PKA under HPHK treatment were significantly increased between 7.21 and 168.01 times, between 10.18 and 114.12 times, and between 32.95 and 134.12 times, respectively (Fig. 5g–i). Moreover, this was significantly higher under HPHK treatment when compared to HPLK in all growth stages.

Sufficient K supply significantly increased AKA, RKA, and TKA in both rice varieties when compared to LK, while HPHK treatment was significantly higher than LPHK in both varieties (Fig. 5j–l). Among these, the AKA, RKA, and TKA in both rice varieties under HPHK treatment significantly increased between 11.31 and 90.30 times, between 5.30 and 124.41 times, and between 11.36 and 75.68 times, respectively.

### PUR and KUR in water samples

**Fig. 6:**
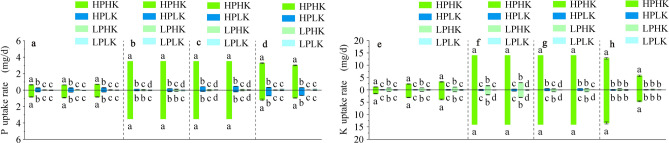
PUR in nutrient solutions (**a**–**d**). Day 10 d to day 30 (**a**), day 40 d to day 50 (**b**), day 60 d to day 70 (**c**) and day 70 d to day 90 (**d**). KUR in nutrient solutions (**e**–**h**). Day 10 d to day 30 (**e**), day 40 d to day 50 (**f**), day 60 d to day 70 (**g**) and day 70 d to day 90 (**h**). TS, HS, FS and MS represent tillering stage, full heading stage, filling stage and maturity stage respectively. The data above the origin of the Y-axis represents SN265, and the data below represents Liaojing 294. The four treatments were HPHK (10 mg/L P and 40 mg/L K), HPLK (10 mg/L P and 2 mg/L K), LPHK (0.5 mg/L P and 40 mg/L K) and LPLK treatment (0.5 mg/L P and 2 mg/L K), respectively. The error bar represents ± standard deviation. Different lowercase letters indicate statistical significance at P < 0.05 level.

The effects of treatments on the PUR and KUR in water samples varied throughout the growth stages of rice. From the 10th to the 30th day, rice plants were between the transplanting and the tillering stages (Fig. 6a,e); from the 40th to the 50th day, rice plants were between the tillering and the full heading stages (Fig. 6b,f); from the 60th to the 70th day, rice plants were between the full heading and the filling stages (Fig. 6c,g); and from the 80th to the 90th day, rice plants were between the filling and the maturity stages (Fig. 6d,h). The PUR in the water samples of both varieties reached a maximum under HPHK treatment, which was significantly different than other treatments. This was followed by HPLK treatment, which was significantly higher than LP, with the exception from the 40th to the 50th day (Fig. 6b).

From the 10th to the 30th day, the PUR of SN265 under HPHK treatment ranged from 0.39 to 0.93 mg/d, and of LJ294 ranged from 0.53 to 1.39 mg/d, with the two rice varieties being significantly increased between 0.77 and 69.25, and between 2.67 and 53.01 times, respectively, when compared to other treatments (Fig. 6a). From the 40th to the 70th day, the PUR of both varieties under HPHK treatment was 3.5 mg/d. Compared with other treatments, the PUR of both varieties under HPHK treatment were significantly increased between 19.03 and 163.32 times, and between 21.54 and 44.63 times, respectively, from the 40th to the 50th day (Fig. 6b), and between 6.94 and 75.59 times, and between 14.35 to 144.83 times, respectively, from the 60th to the 70th day (Fig. 6c). From the 80th to the 90th day, the PUR under HPHK treatment ranged from 3.02 to 3.27 mg/d for SN265, and from 1.01 to 1.11 mg/d for LJ294, with both varieties significantly increased between 9.09 and 81.16 times, and between 0.58 and 150.39 times, respectively, when compared to other treatments (Fig. 6d).

The KUR in water samples of both varieties reached a maximum under HPHK treatment, which was significantly different from other treatments. In addition, the KUR of both rice varieties under LPHK were significantly higher than LK from the 10th to the 50th day; and were higher than LK from the 80th to the 90th day. From the 10th to the 30th day, the KUR under HPHK treatment ranged from 0.99 to 3.17 mg/d for SN265, and from 1.58 to 3.66 mg/d for LJ 294, with both varieties significantly increasing between 1.06 and 42.68 times, and between 3.40 and 155.35 times, respectively, when compared to other treatments (Fig. 6e). The KUR of both varieties under HPHK treatment were 14.00 mg/d from the 40th to the 70th day. Among these, from the 40th to the 50th day, the two varieties under HPHK treatment were significantly increased between 4.00 and 229.64 times, and between 3.70 and 115.38 times, respectively, when compared to other treatments (Fig. 6f). From the 60th to the 70th day, the two varieties under HPHK treatment were significantly increased between 22.00 and 218.78 times, and between 17.20 and 446.28 times when compared to other treatments (Fig. 6g). From the 80th to the 90th day, the KUR under HPHK treatment ranged from 6.05 to 12.44 mg/d for SN265, and from 4.65 to 13.40 mg/d for LJ294, which were significantly increased between 19.69 and 83.06 times, and between 44.27 and 82.48 times, respectively, for both varieties when compared to other treatments (Fig. 6h).

### Root-related indicators

**Fig. 7:**
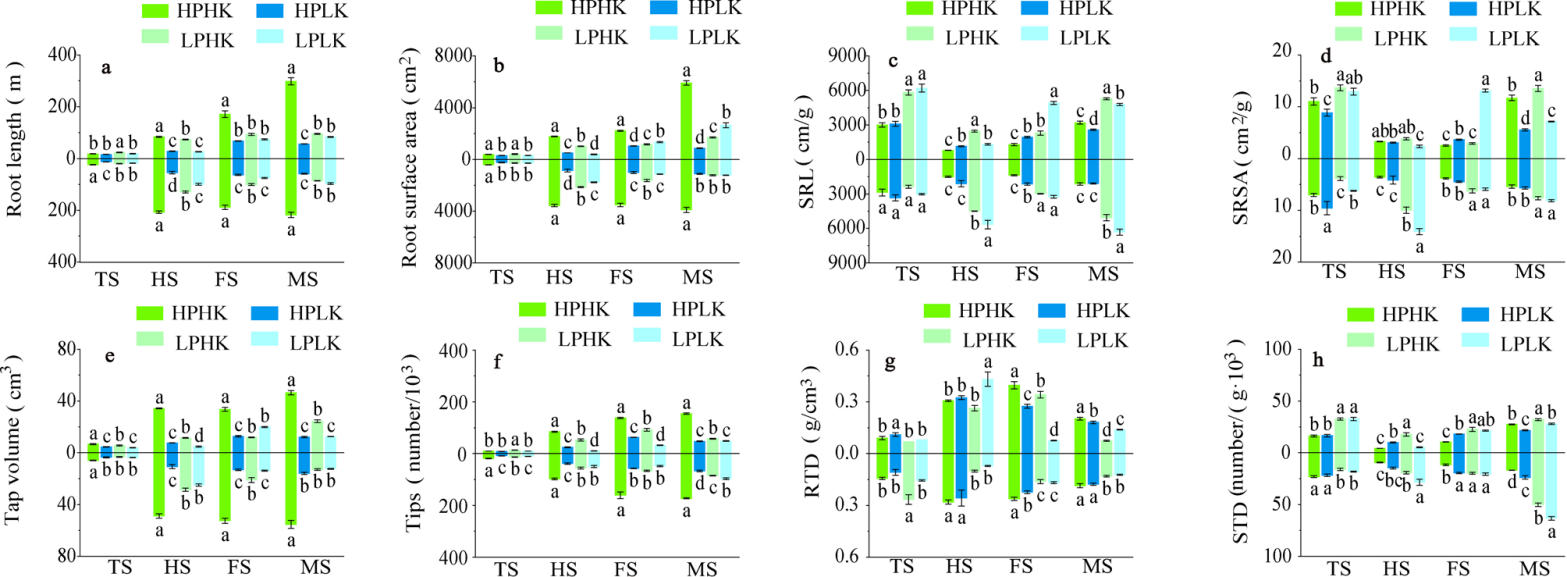
Root-related indicators under different P and K nutrient solutions. Root length (**a**), Root surface area (**b**), SRL (**c**), SRSA (**d**), Total volume (**e**), Tips (**f**), RTD (**g**), and STD (**h**). The data above the origin of the Y-axis represents SN265, and the data below represents Liaojing 294. The four treatments were HPHK (10 mg/L P and 40 mg/L K), HPLK (10 mg/L P and 2 mg/L K), LPHK (0.5 mg/L P and 40 mg/L K) and LPLK treatment (0.5 mg/L P and 2 mg/L K), respectively. The error bar represents ± standard deviation. Different lowercase letters indicate statistical significance at P < 0.05 level.

As shown in Fig. 7, when compared to LP, HPHK treatment significantly increased root length and surface area of the two rice varieties from the full heading to the maturity stages (Fig. 7a,b); significantly decreased the SRL of the two varieties at the filling and maturity stages; and significantly decreased the SRSA of LJ294 from the full heading to the maturity stages (Fig. 7c,d). Compared to LP, root length and surface area were significantly increased between 0.14 and 2.57 times, and between 0.67 and 3.52 times, respectively; and specific root length was significantly decreased by between 0.04 and 73.68% for the two varieties under HPHK treatment. In terms of the SRSA, LJ294 was significantly reduced between 29.50 and 74.66% under HPHK treatment when compared to LP.

Compared to LP, HPHK treatment significantly increased the tap volume and tips from the full heading to the maturity stages of the two rice varieties (Fig. 7e,f); significantly increased the RTD at the filling and maturity stages (Fig. 7g); and significantly decreased the STD from the full heading to the maturity stages of LJ294 (Fig. 7h). Tap volume, tips, and RTD were significantly increased between 0.69 and 6.32 times, between 0.50 and 7.10 times, and between 0.16 and 4.21 times, respectively; while STD was significantly decreased between 40.78 and 73.43%. In addition, HPLK treatment significantly increased the RTD of LJ294 when compared to LP at the full heading and filling stages. Compared with LK, LPHK treatment increased root length, root surface area, and tips of LJ294 from tillering to filling stages (Fig. 7a,b,f).

### Root-related P and K efficiencies

**Fig. 8:**
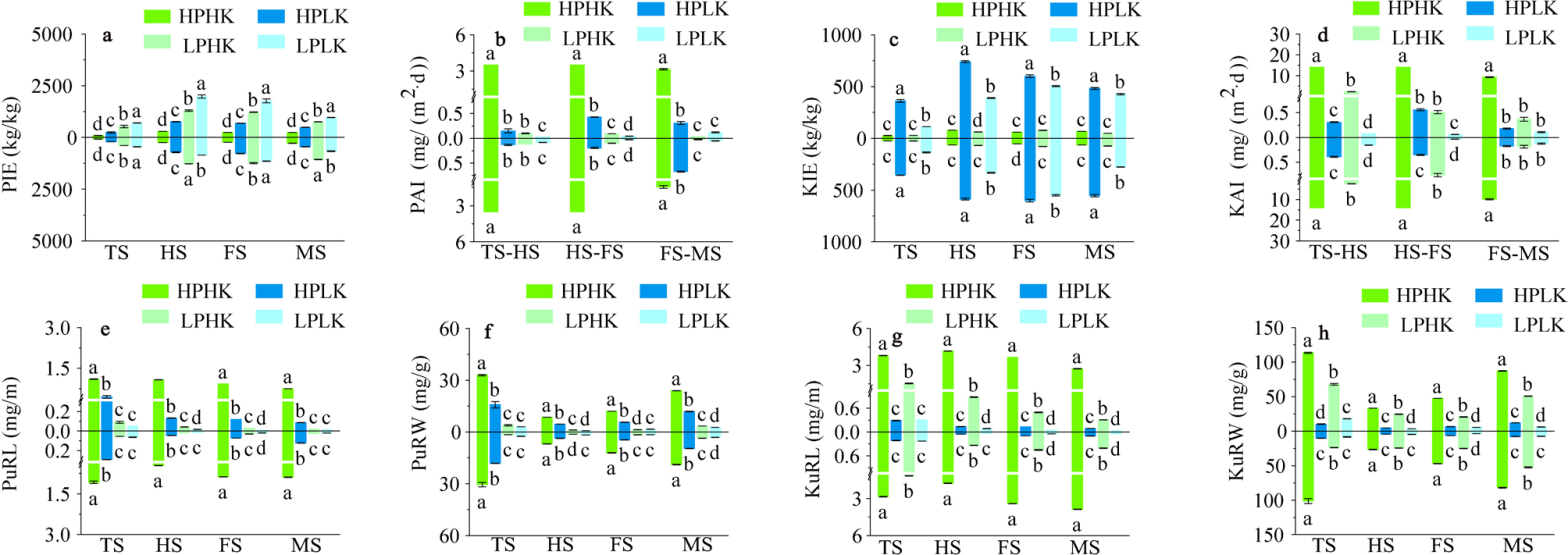
Root-related P and K efficiencies under different P and K nutrient solutions. PIE (**a**), PAI (**b**), KIE (**c**), KAI (**d**), PuRL (**e**), PuRW (**f**), KuRL (**g**), and KuRW (**h**). The data above the origin of the Y-axis represents SN265, and the data below represents Liaojing 294. The four treatments were HPHK (10 mg/L P and 40 mg/L K), HPLK (10 mg/L P and 2 mg/L K), LPHK (0.5 mg/L P and 40 mg/L K) and LPLK treatment (0.5 mg/L P and 2 mg/L K), respectively. The error bar represent ± standard deviation. Different lowercase letters indicate statistical significance at P < 0.05 level.

Sufficient P supply significantly reduced PIE at all growth stages of the two varieties when compared to LP (Fig. 8a); and significantly increased the PAI, PuRL, and PuRW at all growth stages of the two varieties (Fig. 8b,e,f). Meanwhile, compared to HPLK treatment, PIE was significantly lower under HPHK, while the PAI, PuRL, and PuRW were significantly higher under HPHK. Compared to LP, the PIE of the two varieties under HPHK treatment was significantly reduced between 57.89 and 86.74%; while the PAI, PuRL, and PuRW were significantly increased between 25.93 and 130.09 times, between 11.88 and 64.87 times, and between 2.87 and 18.06 times, respectively.

Compared to LK, sufficient K supply significantly reduced KIE at all growth stages of the two rice varieties (Fig. 8c); significantly increased the KAI from the tillering to the full heading stages of the two varieties (Fig. 8d); and significantly increased the KuRL and KuRW at all growth stages of the two varieties (Fig. 8g,h). Moreover, the KAI, KuRL and KuRW were significantly higher under HPHK treatment than in LPHK. Compared to LK, KIE of the two varieties under HPHK treatment was significantly reduced between 76.16 and 93.12%; while the KAI, KuRL and KuRW were significantly increased between 34.90 and 181.53 times, between 11.36 and 75.48 times, and between 4.76 and 13.41 times, respectively.

As shown in Table 3, HPHK treatment significantly increased the GPR, PUE, and PHI, and significantly decreased the PUEb and PPE of both varieties when compared to other treatments. GPR, PUE and PHI of SN265 under HPHK significantly increased by 0.47 to 1.27 and 0.68 to 1.06 times, 0.81 to 7.58, while LJ294 by 0.58 to 6.24 times and 5.41% to 48.39% and 37.42% to 78.62%, respectively. The PUEb of both varieties significantly decreased between 49.41 and 78.28% times, and between 40.82 and 75.40%, respectively, and the PPE significantly decreased between 32.37 and 56.31%, and between 40.54 and 51.63%, respectively. Compared to other treatments, HPHK significantly increased the KUE and significantly decreased the KTE of the two varieties. Among these, the KUE of SN265 and LJ294 under HPHK treatment was significantly increased between 0.55 and 7.91 times, and between 0.90 and 8.47 times, respectively. Compared to LK, HPHK significantly increased the KHI of SN265, while sufficient K supply significantly increased the GKR of the two varieties, and significantly decreased the KUEb and KPE. In terms of KAE, the order of treatments was: HPLK > HPHK > LPLK > LPHK. These results indicated that HPHK treatment mainly increased the GPR, PUE, PHI, and KUE of the two varieties, and had a negative effect on the PUEb, PPE, and KTE when compared to other treatments.

**Table 3:** Effect of treatments on yield-related P (K) efficiency of the two varieties.

Indicators	SN265				LJ294			
	HPHK	HPLK	LPHK	LPLK	HPHK	HPLK	LPHK	LPLK
GPR	106.47 ± 1.28 a	72.67 ± 4.87 b	48.23 ± 4.55 c	46.80 ± 2.62 c	104.95 ± 2.66 a	62.42 ± 1.76 b	50.94 ± 2.46 bc	57.09 ± 2.86 c
PUEb	288.63 ± 2.93 d	570.51 ± 6.83 c	1047.21 ± 7.04 b	1329.08 ± 5.91 a	331.56 ± 6.38 d	560.26 ± 4.21 c	1347.65 ± 17.64 a	982.65 ± 2.76 b
PAE	66.40 ± 0.57 a	11.45 ± 0.83 b	82.54 ± 8.59 a	64.55 ± 4.04 a	58.95 ± 1.07 b	13.72 ± 0.46 c	77.04 ± 2.85 a	53.12 ± 3.33 b
PPE	93.94 ± 1.12 c	138.90 ± 9.56 b	211.47 ± 21.95 a	215.03 ± 12.23 a	95.41 ± 2.46 c	160.47 ± 4.64 b	197.24 ± 9.84 ab	176.06 ± 9.11 a
PUE	70.69 ± 0.30 a	8.24 ± 0.11 d	39.03 ± 0.04 b	30.00 ± 0.22 c	61.81 ± 0.53 a	8.54 ± 0.13 d	39.11 ± 0.49 b	30.15 ± 0.77 c
PTE	35.58 ± 2.28 b	46.77 ± 1.79 a	32.13 ± 1.18 b	16.35 ± 1.20 c	43.79 ± 0.49 a	37.15 ± 1.61 b	14.87 ± 0.57 d	30.54 ± 0.71 c
PHI	74.80 ± 0.32 a	53.54 ± 0.83 c	50.41 ± 0.62 d	70.96 ± 1.13 b	64.10 ± 0.47 a	46.65 ± 0.88 b	35.89 ± 1.13 c	46.35 ± 1.20 b
GKR	388.80 ± 3.54 b	73.05 ± 3.78 c	714.83 ± 63.24 a	104.94 ± 5.19 c	454.97 ± 10.76 b	51.37 ± 0.87 d	736.34 ± 28.28 a	135.80 ± 9.21 c
KUEb	79.05 ± 1.28 c	566.56 ± 10.36 b	70.59 ± 0.97 c	592.31 ± 2.65 a	76.49 ± 1.80 c	680.56 ± 8.72 a	93.22 ± 2.66 c	414.28 ± 12.13 b
KAE	16.60 ± 0.14 b	57.25 ± 4.14 a	1.03 ± 0.11 c	16.14 ± 1.01 b	14.74 ± 0.27 b	68.57 ± 2.30 a	0.96 ± 0.04 c	13.28 ± 0.8294 b
KPE	25.72 ± 0.24 c	137.66 ± 7.49 a	14.23 ± 1.37 c	95.77 ± 4.83 b	22.01 ± 0.52 c	194.80 ± 3.36 a	13.62 ± 0.54 c	74.29 ± 4.85 b
KUE	64.53 ± 0.24 a	41.51 ± 0.77 b	7.24 ± 0.06 d	16.83 ± 0.23 c	67.00 ± 0.38 a	35.19 ± 0.72 b	7.07 ± 0.10 d	17.88 ± 0.07 c
KTE	15.82 ± 2.68 d	36.94 ± 1.94 b	51.89 ± 3.51 a	24.68 ± 1.94 c	19.78 ± 2.70 c	61.17 ± 0.95 a	41.78 ± 2.00 b	56.52 ± 2.77 a
KHI	55.36 ± 0.58 a	46.35 ± 1.36 b	52.04 ± 2.52 a	31.38 ± 1.58 c	56.35 ± 0.74 b	63.19 ± 0.54 a	47.19 ± 0.50 d	50.94 ± 1.09 c

The four treatments were HPHK (10 mg/L P and 40 mg/L K), HPLK (10 mg/L P and 2 mg/L K), LPHK (0.5 mg/L P and 40 mg/L K) and LPLK treatment (0.5 mg/L P and 2 mg/L K), respectively. Different lowercase letters indicate statistical significance at P < 0.05 level.

## Discussion

Sufficient P supply (45 ppm) can effectively increase the DMabove and DMroot in wheat, while low K significantly reduces the DMabove and DMroot in cotton^38^. Suitable applications of P or K can reduce the root–shoot ratio of cotton or rice plants^39,40^. In our study, compared with other treatments, HPHK significantly increased dry matter accumulation in stem sheath, leaf, panicle and root of both varieties and significantly decreased root–shoot ratio during main growth stages (Figs. 2d, 3a–c,e). Suitable P application can significantly increase grain N concentration and promote grain N and P accumulation^41^. K application can promote the increase of P and K concentration in potato tubers^42^. In our experiment, HPHK treatment significantly increased the P concentration in the stem sheath, leaves, aboveground parts, and roots of the two rice varieties during the main growth stages when compared to LPHK and LPLK (Fig. 4a,b,d,e), and significantly increased the P concentration in the panicles from the filling to the maturity stages (Fig. 4c). Compared to HPLK and LPLK treatment, HPHK significantly increased the K concentration in the stem sheath, leaves, aboveground parts, and roots of the two rice varieties during the main growth stages (Fig. 4f,g,i,j), and significantly increased the K concentration in the panicles from the filling to the maturity stages (Fig. 4h).

With sufficient P supply, the balance between the indices of potato root morphology is conducive to roots having a stronger P absorption^43^. More developed roots, such as larger root weight and root volume, can promote the uptake of K from the environment by rice roots^44^. In our experiment, HPHK treatment significantly increased P (K) accumulation in the stem sheath, leaves, aboveground parts, and roots of the two rice varieties during the main growth stages when compared to LP (K) (Fig. 5a,b,d,e,g,h,j,k), and significantly increased panicle P (K) accumulation from the full heading to the maturity stages (Fig. 5c,i). This indicated that HPHK treatment can maintain a balance in root morphology indicators while increasing root length, root surface area, total root volume, and number of root tips, thus promoting the root’s P and K uptake. Additional accumulation of P and K in roots was further transferred to various organs in the ground to meet the P and K demands for plant growth. In addition, HPLK treatment increased the plant P concentration and accumulation at the maturity stage when compared to LP, while LPHK treatment increased the plant K concentration and accumulation when compared to LK levels. This showed that the P and K concentrations in the environment visibly affected the P and K concentration and accumulation of plants^45,46^.

Suitable P application can effectively promote root metabolism and affect leaf and root ACP activity after flowering^47^. In addition, suitable K application can improve root morphology and physiological characteristics^48^. In this experiment, HPHK treatment significantly increased the RA of the two rice varieties at the main growth stage when compared to LP (Fig. 2e), and significantly decreased the ACP activities of leaves and roots (Fig. 2a,b). Stronger root activity can promote the uptake of nutrients by plant roots, thereby increasing rice yield^49^.

Although the increase of ACP activity indicated that plants were more adaptable to stress, HPHK treatment itself was not considered a stressful state, and therefore it significantly reduced ACP activity. This is similar to findings reported by Barrett-Lennard et al. where P-deficiency increases ACP activity in wheat leaves, while P supplementation decreases it^50^. In addition, the amplitude of change in ACP activity and RA under LPHK and LPLK treatments were significantly smaller than those at HP levels, with HPLK treatment significantly inhibiting root growth. This indicated that the effect of P on regulating RA and plant ACP activity was significantly larger than that of K. Under sufficient P supply, K-deficiency can severely inhibit root growth. Therefore, we believe that the reasonable supply of K under a sufficient P supply is beneficial to improve root configuration and physiological characteristics.

The effects of P and K supply on root morphology were different. P mainly played a role in lateral root development, while K mainly played a role in root length and quantity^51,52^. In the root splitting test, the co-supply of P and K (applied together) was more beneficial to the root growth of wheat, and promoted plant P and K uptake when compared to the separate supply of P and K (supplied separately on both sides)^53^. This indicates that P and K may have a synergistic effect on plant growth and nutrient absorption under sufficient supplies of P and K. In addition, the effect of co-application of P and K is evidently greater than the single application of P and K. In this experiment, compared to LPHK and LPLK treatments, HPHK significantly increased the total root length (Fig. 7a), total surface area (Fig. 7b), total root volume (Fig. 7e) and number of root tips (Fig. 7f) in the two studied rice varieties from the full heading to the maturity stages, effectively improving root configuration. Compared to LPHK treatment, HPHK improved root morphology mainly due of P. Compared to LPLK treatment, HPHK improved root morphology mainly due to the synergistic effects of P and K interactions. In addition, LPHK and LPLK treatment resulted in negligible differences in root morphology. This shows that the role of P in regulating root morphology is notably greater than that of K. Under sufficient P supply, the sufficient supply of K can maximize the synergistic effect of P and K interactions. However, in the case of a P-deficient supply, the promoting effect of a sufficient K supply on root structure cannot compensate for the inhibiting effect caused by low P. In this case, P and K interactions had antagonistic effects on regulating root configuration. Therefore, we believe that although the importance of K in regulating root configuration is much lower than that of P, the reasonable allocation of P and K ratios is necessary to fully benefit from the synergistic effects of P and K on root configuration.

Low P supply significantly increased cotton root SRL, STD, SRL and SRSA and root hair density^54,55^. Meanwhile, low K supply significantly inhibits root growth and root length density^56^. In this experiment, from the filling to the maturity stages, HPHK treatment significantly reduced the SRL compared to LPHK and LPLK (Fig. 7c) and significantly increased the RTD (Fig. 7g) of the two rice varieties, but had no obvious effect on the SRSA (Fig. 7d) and STD (Fig. 7h). The decrease of SRL and the increase of RTD indicate that the root length and total root volume under HPHK treatment coincide with higher root dry weights. However, in order to adapt to a stressful environment under LP, the growth rate of root length and total root volume may be higher than that of root dry matter accumulation under low P when compared to HPHK treatment. This is similar to the results of Lozano et al.^57^, which indicate that in order to adapt to a drought stress environment, plant RTD decreased significantly, and promoted plant nutrient uptake. In addition, although the root surface area and root tip number under HPHK treatment were significantly higher than the LP level, these may not coincide with the accumulation pattern of root dry weight at different growth stages, resulting in a response law of SRSA (ratio of root surface area to root dry weight) and STD (ratio of root tip to root dry weight) on P and K supply.

With sufficient supply of P and potassium, the root morphology and spatial distribution of rice were optimized during the whole growth period, ensuring a relatively stable root–shoot ratio. Many studies have shown that low P can significantly improve root parameters of rice. However, in this study, although the roots of plants under long-term stress had improved to adapt to stress, that is, the roots extended downward, the roots were still seriously damaged. Long-term lack of external P supply resulted in a decrease in root activity (Fig. 2e). Therefore, the adaptive changes of root growth of rice plants under low P stress could not compensate for the harm caused by P deficiency in the environment. This indicated that the ability of rice plants to adapt to low P stress under P deficiency was limited, and it depended on the degree of P inhibition in the environment. This may be related to the changes of root hormones, especially IAA and ethylene, and related gene expression about by low P^58,59^. In addition, although the effect of K on roots was significantly smaller than that of P, under sufficient P supply, HPLK treatment significantly reduced root-related parameters (Fig. 7a,b,e,f), indicating that low K also had a certain inhibitory effect. Under low potassium, root activity decreased significantly, indicating serious root damage, which may be related to Na^+^/K^+^ imbalance in cells, K channel protein-related gene expression and root anatomical structure^60–62^. However, in this study, there was a lack of research on the effects of P and K on hormone synthesis in rice roots, K^+^ channel protein and other signaling pathways and related genes.

Suitable application of P and K can significantly affect the grain yield and yield composition of rice^63^. In our experiment, HPHK treatment significantly increased the effective panicle number, grain number per panicle, and rice yield of both rice varieties when compared to other treatments (Fig. 1b,d,e). This is similar to a study by Fageria et al. that indicated that suitable P application can effectively improve yield components, such as ear density and 1000-grain weight of rice^64^. In addition, HPHK treatment significantly increased the GPR, PHI, PUE, and KUE in the two rice varieties, and significantly reduced the PUEb, PPE, KTE, and PIE (KIE) when compared to other treatments (Table 3; Fig. 8a,c). P application can regulate the efficiency of P utilization for the GPR, PAE, PPE and PUE by increasing the P accumulation in plants^65,66^. Therefore, we believe that sufficient P and K supply promotes the increase of P and K accumulation in the panicle and whole plant and grain yield, which significantly affects the associated P and K utilization efficiency. However, a reasonable allocation of P and K supply ratios is necessary to maintain plant nutrient balance, thereby improving the relationship between the efficiency of P and K. This topic still warrants further investigation.

The increase of PuRL and PuRW was beneficial to enhance P uptake^67,68^. A higher KuRL indicates that the plant has a better ability to acquire K from its roots^69^. Plants may improve their K absorption efficiency by improving their root configuration and increasing their KuRW^70^. In our experiment, sufficient P (K) supply significantly increased the PAI (KAI), PuRL (KuRL), and PuRW (KuRW) at all growth stages of both rice varieties when compared to LP (K) levels. In addition, the above indexes all reach a maximum under HPHK treatment (Fig. 8b,d,e–h). This may be due to the sufficient supply of P and K enhancing the ability of roots to obtain P and K from the environment by improving root configuration, promoting P and K uptake and substance accumulation of plants, and increasing the PAI (KAI), PuRL (KuRL), and PuRW (KuRW) of plants. HPLK treatment is superior to LPHK and LPLK in regard to P efficiency. Under K-efficiency, LPHK treatment is superior to HPLK and LPLK treatments. These results indicated that the changes of PAI (KAI), PuRL (KuRL), and PuRW (KuRW) were mainly determined by the concentration of P and K in the nutrient solution and plant P and K absorption. The promotion of the PAI (KAI), PuRL (KuRL), and PuRW (KuRW) by a sufficient supply of P or K was greater than the inhibition caused by deficient K or P levels. In addition, the PUR (KUR) in the nutrient solution can indicate the ability of the plant to absorb P and K from the nutrient solution. Therefore, in this experiment, with the enhancement of the PAI (KAI), the supply of sufficient P and K significantly increased the PUR (KUR) in each growth stage of the two rice varieties. This further proved that the effects of a sufficient supply of P and K on enhancing plant P and K absorption was significantly superior to other treatments. In nutrient solutions and soil environments, the effects of the same treatment are not exactly the same. In soil, the absorption of rice root nutrients not only comes from the supply of fertilizers in the soil, but also may be affected by the physical and chemical properties of the soil itself, soil pH, and microbial activities^71^. In the hydroponics experiment, the external P and K absorption of rice plants came from the nutrient solution, so the concentration of P and K in the nutrient solution directly determined the plant growth. In this paper, P and K supply can directly reflect the effect of P and K on the plant itself. HPHK supply had obvious synergistic effect on root efficiency and yield-related efficiency, which enhanced the external ability of plants to obtain P and potassium, and thus increased rice yield.

**Fig. 9:**
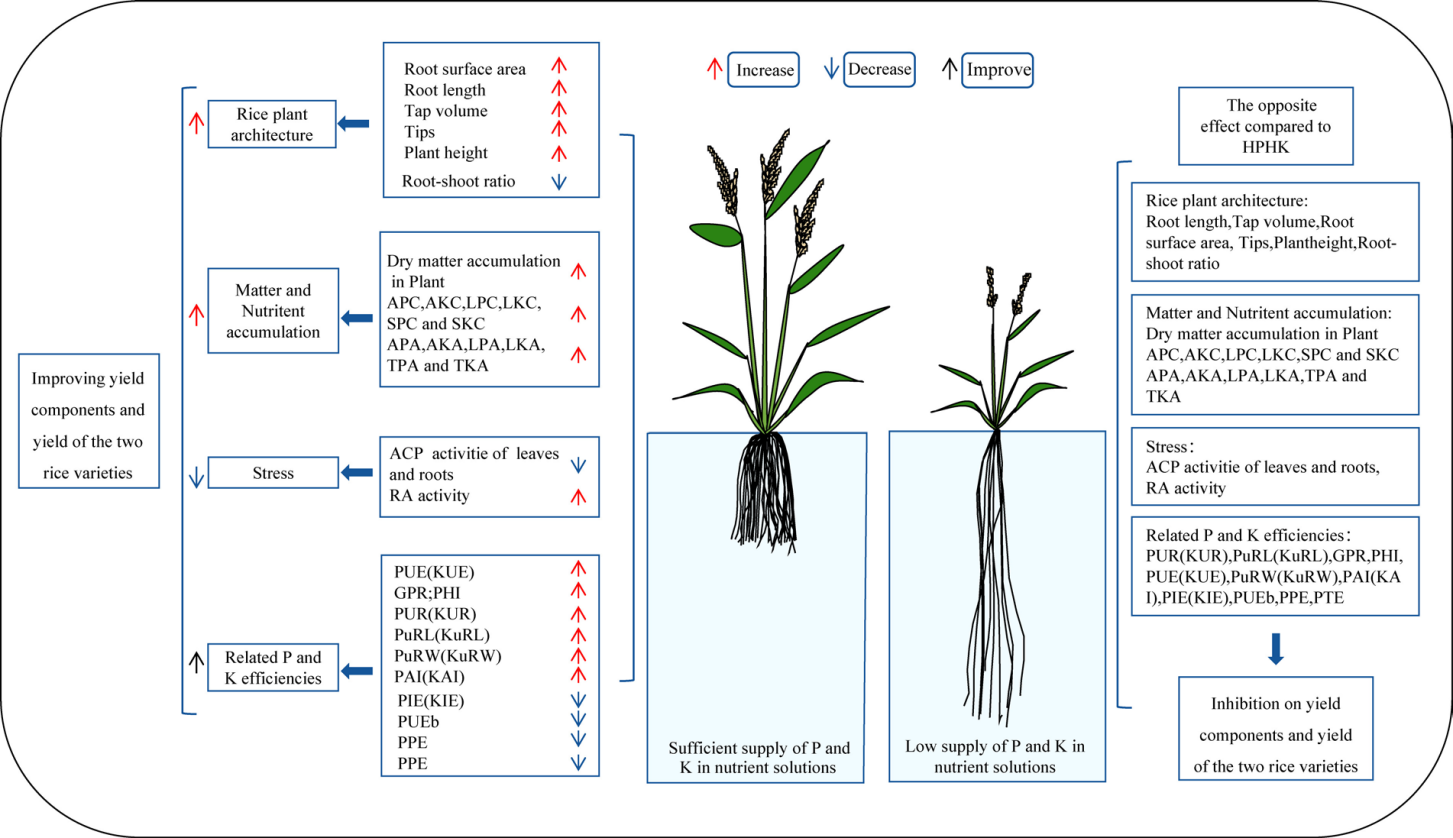
Summary model of the two varieties under HPHK and LPLK.

As shown in Fig. 9, taking HPHK and LPLK as examples, the overall response model of the two varieties to relevant indicators was drawn. Sufficient supply of P and K had obvious synergistic effects on plant height, leaf development, effective panicle number, root morphology and plant architecture of the two varieties. Under low P and low K supply, the plants of the two varieties were short, the number of leaves and effective panicle was less, and the roots extended downward to adapt to the stressed environment, and the root activity decreased significantly. Sufficient supply of P and K significantly increased the dry weight and above-ground P (K) concentration and P (K) accumulation of each part of the two varieties during the main growth period. Low P and low K supply showed obvious antagonistic effect. In addition, the plants under HPHK were not stressed, and showed less ACP activity and stronger root activity. Plants under LPLK showed long-term low P and stress, and ACP activity of leaves and roots increased significantly in order to adapt to stress environment. In terms of P and K utilization efficiency, HPHK can effectively improve the efficiency of PUR (KUR), PuRL (KuRL), PuRW (KuRW), PUE (KUE) and PAI (KAI). LPLK showed the opposite pattern.

In summary, HPHK has certain advantages in plant architecture, dry matter and P and K accumulation, reducing stress degree and improving related P and K utilization rate in the “root-yield-nutrient solution” system, so as to effectively improve rice yield composition and promote rice yield increase.

## Conclusion

Sufficient supply of P and K can optimize plant architectures (plant height, root–shoot ratio), improve yield components (effective panicle number, grain number per panicle) and root morphological structures (total root length, total surface area, total root volume, and number of root tips) of the two investigated rice varieties. Sufficient supply of P and K can also promote plant dry matter accumulation and nutrient absorption, thereby increasing the PUR (KUR) in nutrient solutions and the PAI (KAI), PuRL (KuRL), PuRW (KuRW), GPR, PUE, PHI, and KUE in plants. In addition, lower ACP activity and higher RA indicated that HPHK treatment was less stressful to plants. HPHK treatment can improve root configuration and yield components, increase P and K absorption and associated P and K efficiency, and reduce the degree of stress in plants, thereby jointly maintaining normal plant growth and nutrient absorption, and promoting increases in rice yield. Moreover, the effects of K on these indicators occur at a lower magnitude when compared to P.

## Data Availability

The raw data supporting the conclusions of this article are available from the corresponding author upon reasonable request.
